# Education Scholarship and its Impact on Emergency Medicine Education

**DOI:** 10.5811/westjem.2015.9.27355

**Published:** 2015-10-22

**Authors:** Jonathan Sherbino

**Affiliations:** McMaster University, Department of Emergency Medicine, Hamilton, Ontario, Canada

## Abstract

Emergency medicine (EM) education is becoming increasingly challenging as a result of changes to North American medical education and the growing complexity of EM practice. Education scholarship (ES) provides a process to develop solutions to these challenges. ES includes both research and innovation. ES is informed by theory, principles and best practices, is peer reviewed, and is disseminated and archived for others to use. Digital technologies have improved the discovery of work that informs ES, broadened the scope and timing of peer review, and provided new platforms for the dissemination and archiving of innovations. This editorial reviews key steps in raising an education innovation to the level of scholarship. It also discusses important areas for EM education scholars to address, which include the following: the delivery of competency-based medical education programs, the impact of social media on learning, and the redesign of continuing professional development.

## INTRODUCTION

Since a massive reorganization more than a century ago, medical education in North America has remained largely unchanged.^1^ However, increasing calls from the public for accountable care have been a lever to influence the reorganization of medical education. Changes in medical education include increasing attention to patient outcomes and patient safety,^2^,^3^ restriction of resident duty hours,^4^ increasing standards of physician certification,^5^,^6^ and the reorganization of training programs around competency frameworks.^7^,^8^

Within emergency medicine (EM) the clinical teaching environment is becoming increasingly challenging with frequent interruptions, increasing patient volumes and complex patient presentations.^9^,^10^ The cohort of learners being trained in emergency departments (EDs) is also rising through the expansion of EM training programs and the demand for off-service resident exposure to EM.^11^ Finally, the competencies required of EM physicians are expanding as the science and practice of EM grows.

Within the context of this rapidly changing clinical and educational environment it is important to acknowledge that how a person learns remains constant. While education science and cognitive psychology is a growing field, these disciplines are simply gaining an increasing understanding of the established learning processes. Medical education is being forced to change to meet public, institutional, and clinical demands. The challenge for learners and teachers is to ensure that changes to education systems are appropriate, efficient and lead to best patient outcomes.

During this key period in medical education, education scholarship (ES) is emerging as an important academic pursuit to influence change. The purpose of this article is to define ES, articulate the different types of practice of education scholars and suggest areas of scholarship that will be influential for EM education.

## DEFINING EDUCATION SCHOLARSHIP

Education research has an established, although narrow and specific, role in academic medicine. As with all types of research, education research uses the scientific method where observations inform testable hypotheses via a perpetually spiraling cycle. The outcomes of experiments lead to dynamic, explanatory theories. Recognizing the simplification of this description, education research is principally focused on discovery.

Nearly three decades ago, Boyer articulated a vision for academics that suggested the research of discovery was not an exclusive goal.^12^ Rather, integration (i.e., synthesizing and making connections from difference fields or disciplines), application (i.e., applying theory to practice) and teaching (i.e., determining best practices for learning) are equally valid goals for academic physicians.^13^ In this framework, scholarship encompasses discovery, integration, application and teaching. The significance of this work is that it broadens the career and academic options of EM educators by validating contributions not typically defined by “research.”

**Figure 1:**
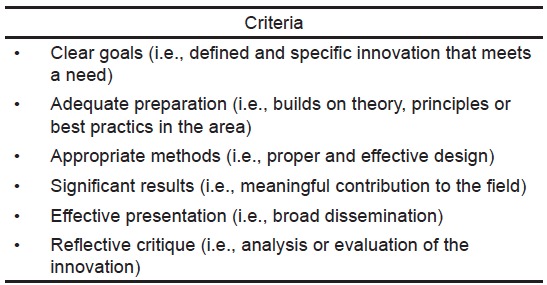
Criteria for assessing an education innovation as scholarship.^17^

Building on this and other work,^14^,^15^ the Canadian Association for Medical Education provides a definition of ES that addresses previous difficulties with discretely categorizing the above concepts. ES is “an umbrella term which can encompass both research and innovation in health professions education. Quality in [ES] is attained through work that is peer-reviewed, publicly disseminated and provides a platform that others can build on.”^16^ Again, this definition emphasizes that an important contribution to the mission of teaching hospitals, universities, and other academic organizations is the development of education innovations (e.g. novel trainee selection process, teaching method, assessment instrument, curriculum, faculty development initiative etc.), many of which provide solutions to the challenges currently facing EM education. Importantly, this definition incorporates the criteria suggested by Glassick^17^ as necessary to adjudicate education work, especially innovations, as scholarly. (See Figure 1 for details.) The Academic Section of the Canadian Association of Emergency Physicians has endorsed the above definition of ES as a means to both promote and support education work.^11^

Scholarly innovation is partially analogous to knowledge translation. Evidence-based medicine has become one of the most influential forces in clinical medicine^18^ because knowledge translation has allowed bench research to inform clinical practice.^19^ In a similar fashion, scholarly innovation, influenced by education research, can broadly inform clinical education. The challenges facing EM education, as indicated above, require broad and coordinated attention. If each training program perpetually “re-invents the wheel” to address an education challenge, the delivery of EM education will be haphazard and variable. However, if the EM community promotes ES, rigorous solutions to common challenges can be disseminated, archived and built upon, allowing for progressive advancement of the field.

## THE INFLUENCE OF TECHNOLOGY ON EDUCATION SCHOLARSHIP

The standards of scholarship are well established, but it is the ascendance of digital technologies that permit ES, particularly innovations, to take off. For example, all scholarship builds on previous theory, principles or best practices,^11^ which requires a formalized search of the literature to ensure that scholarship is properly grounded and situated to advance the field. Traditionally, a literature search has meant the use of established databases (e.g. PubMed, Embase, ERIC, etc.) and medical subject headings (MEsH), none of which are well structured to support education searches. Moreover, a portion of valuable education literature is gray literature “which is produced on all levels of government, academics, business and industry in print and electronic formats, but which is not controlled by commercial publishers.”^20^ The sophisticated algorithms of web search engines (e.g., Google Scholar) allow the identification of education innovations in the gray literature that previously would not have been discovered by traditional literature search methods. This new process allows for more effective knowledge sharing and synthesis.

Peer review, the second key component of scholarship, has traditionally involved a small (e.g., two to five) group of content experts. As any editor or author knows, the quality of peer review can be quite variable and subjective, owing in part to the size of the peer-review sample. With the inclusion of comment and discussion features on journal websites and the prominence of social media-based discussion via blogs, microblogs (e.g., Twitter) and networking websites (e.g., Facebook), post-publication review of ES has rapidly developed. This crowd-sourced review process can promote a more transparent (provided reviewers identify themselves) and rigorous (provided the sample is sufficiently large to achieve saturation and has appropriate representation of content expertise) review.^21^

Finally, digital technologies provide new platforms for dissemination beyond traditional print journals and conference proceedings. Disseminating and archiving research findings or innovations are the final key criteria for scholarship. This step allows the field to advance through exposure to new work, provides the opportunity for peer review and informs future scholarship. Certainly, traditional journals have started to acknowledge that education innovation is important for inclusion (e.g., *WestJEM* Educational Advances submission category). However, open-access publishers (e.g. the Winnower, thewinnower.com), portals (e.g., MedEdPORTAL, mededportal.org), websites (e.g., Academic Life in EM Medical Education in Cases series, aliem.com/medic/), blogs (e.g. *iTeachEM*, iteachem.net), podcasts (e.g. *KeyLIME* - Key Literature in Medical Education https://itunes.apple.com/ca/podcast/keylime/id594247091?mt=2), and mobile apps (e.g., CanMEDS Springboards for Emergency Physicians, itunes.apple.com/ca/app/canmeds-springboards-for-emergency/id555109611?mt=8) are all examples where digital technology provides alternate platforms to print media for disseminating and archiving innovations. In many of these instances the influence of the free, open-access medical education (FOAM) movement, developed within the EM community, improves audience accessibility when compared with print (i.e., subscription-based) journals.^22^

## DEVELOPING EDUCATION SCHOLARS

There are three general categories of education scholars. The original education scholars are education researchers in the traditional mold. With graduate level training, protected time and academic trajectories based on successful grant applications and publication records, this group follows a traditional academic pathway, where content expertise is education science.^23^

**Figure 2:**
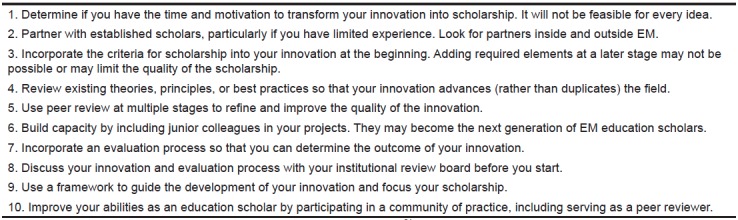
Key steps to transform an education innovation into scholarship.^24^ *EM*, emergency medicine

The second, and largest, group is frontline EM teachers who are invested in a particular project and want to take the necessary steps to raise their work to the standards of scholarship. Unlike education research, the barrier to education innovation is low; thus, support for these “grassroots” efforts can yield significant returns. When an education innovation is raised to the standard of scholarship the teacher benefits from increased academic recognition. For the EM education community, when education innovations are appropriately developed and widely disseminated common needs are often addressed, preventing reduplication of effort, and decreasing the collective work of EM educators. Ten key steps to raise an education innovation to the standard of scholarship are detailed in Figure 2.^24^

However, to address the complex, multi-faceted practical issues currently facing EM education will require a dedicated group of education scholars who contribute in a sustained and directed, rather than opportunistic, fashion. This third group of clinician educators, in contrast to the other two groups, is committed to clinical practice to ensure familiarity with the issues facing education programs, grounded in education theory, and regularly producing scholarship (particularly innovations) that systematically addresses the complex needs of the EM education community.^25^ A clinician educator occupies a unique space between education researchers and frontline teachers, formally trained but focused on frontline problems that require innovations. This (presumably smaller) group requires three key elements for success.

First, formal, but practical, training allows for grounding in education theory that informs scholarship. Akin to the research graduate training necessary for a career as an education researcher, a medical education fellowship orients a dedicated clinician educator to best practices and informing theories when designing an innovative curriculum or assessment instrument. The core concepts required of such fellowships for EM education scholars have been well articulated.^26^–^28^

Second, dedicated clinician educators cannot excel while working in isolation. A community of practice, defined as “the collaborative, informal networks that support professional practitioners in their efforts to develop shared understandings and engage in work-relevant knowledge building,”^29^ is an important element of success. Communities of practice are collectively engaged in the creation of new ideas and innovations. Communities of practice connect education scholars across institutions, leveraging collective analysis of a problem to create an innovative solution. Participation involves more than networking or exchanging data. Junior members can be fostered via legitimate peripheral participation, progressively developing abilities that allow greater contribution over time.^30^ Thus, EM teachers who approach ES as opportunistic (i.e. “one-off”) can be progressively engaged to more regularly contribute to the collective issues facing the EM education community.^31^

Finally, in the same manner as successful researcher, successful clinician educators require institutional support and resources. The important step here is articulating to funding agencies and institutional leadership the educational value and academic legitimacy of theory-informed, peer-reviewed, publically disseminated innovations. In one national environmental scan of institutional support for ES, it was found that only 50% of institutions specifically included education innovation as an acceptable avenue for academic promotion.^16^ Another North American study showed that promotion based on ES was less valued than clinical research.^32^ If the quality of medical education research correlates with the amount of funding, than possibly the same may be true for the quality of medical education innovation.^33^ Bandiera et al. articulate the key elements required to support a culture of ES, including protected time, infrastructure funding, reward models for productivity, and widely accepted metrics (especially alternative metrics to traditional markers) to measure impact and outcomes.^34^

## KEY EM EDUCATION SCHOLARSHIP AREAS

Recognizing that an issue explored by an education scholar will be influenced by the specifics of personal interest and local need, there are a number of emerging topics relevant to education that deserve attention from the EM education community.

### Delivery of Competency-based Medical Education (CBME) Programs

CBME organizes the delivery of EM education based on the abilities (i.e. competencies) required of graduates.^35^ The Accreditation Council for Graduate Medical Education/American Board of EM Milestones Project,^7^ the Association of American Medical Colleges Core Entrustable Professional Activities for Entering Residency,^36^ and the CanMEDS 2015 Framework^8^ are all CBME initiatives. Presumably, they have been implemented to meet the contemporary challenges of medical education. However, a lot of work is still required to make these initiatives functional. How will work-based assessment be designed in a valid fashion?^37^ How will personalized learner advancement, based on completion of milestones, be balanced against the service needs of pre-established rotations? Will accreditation continue as a function of process (e.g. number of rotations completed, availability of hospital resources etc.) or outcomes (e.g. quality of patient care)?^38^ Program directors, educators and front-line teachers equally have an opportunity to collectively contribute to the innovations that will make CBME work.

### Impact of Social Media on Learning

Technology has permitted individuals from around the world to collectively share, curate and develop medical education resources.^39^ The rapid adoption of social media and FOAM is highly influential in EM education. For education scholars there are numerous issues that require attention. For example, how is quality determined in a non-hierarchical, immediate-publication environment?^40^,^41^ How can alternative metrics be used to demonstrate the impact of scholarly innovations?^42^ How can just-in-time digital education resources be used to optimize bedside clinical care?^43^,^44^

### Redesign of Continuing Professional Development/Continuing Medical Education

A third key EM ES topic addresses the ongoing maintenance of competence of physicians in practice. With the rapid advancement of medical science, the learning curves of physicians in practice can no longer be assumed to maintain as a plateau, rather, continued growth should be anticipated.^45^ Moreover, while traditional approaches to continuing professional development (CPD) have focused on the individual,^46^ emerging constructs view the patient care team (i.e. microsystem) as the unit of intervention.^47^ For an EM education scholar, how is ongoing learning assessed and certified in practice? What innovations promote team-based, in-situ learning? Is there a role for maintenance of certification of a team?

## CONCLUSION

As medical education in North America reorganizes and EM practice becomes increasingly complex, the need for ES to provide solutions and inform best practices grows. ES, including both research and innovation, is informed by theories and principles, peer reviewed and disseminated and archived for others to use. Distinct from education researchers and frontline teachers, there is a need for clinician educators to systematically develop innovations that address the common, practical needs of the education community. Finally, important areas for EM education scholars to address include the delivery of competency-based medical education programs, the impact of social media on learning, and the redesign of continuing professional development.

## References

[1] Irby DM, Cooke M, O’Brien BC (2010). Calls for reform of medical education by the Carnegie Foundation for the Advancement of Teaching: 1910 and 2010. Acad Med.

[2] Kalet AL, Gillespie CC, Schwartz MD (2010). New measures to establish the evidence base for medical education: identifying educationally sensitive patient outcomes. Acad Med.

[3] The Safety Competencies Steering Committee (2008). The Safety Competencies: Enhancing Patient Safety Across the Health Professions.

[4] National Steering Committee on Resident Duty Hours (2013). Fatigue, Risk and Excellence: Towards a Pan-Canadian Consensus on Resident Duty Hours.

[5] Nasca TJ, Philibert I, Brigham T (2012). The Next GME Accreditation System - Rationale and Benefits. N Engl J Med.

[6] Hawkins RE, Lipner RS, Ham HP (2013). American Board of Medical Specialties Maintenance of Certification: theory and evidence regarding the current framework. J Contin Educ Health Prof.

[7] Beeson MS, Carter WA, Christopher TA (2013). The development of the emergency medicine milestones. Acad Emerg Med.

[8] Frank JR, Snell LS, Sherbino J (2014). Draft CanMEDS 2015 Physician Competency Framework – Series III.

[9] Chisholm CD, Collison EK, Nelson DR (2000). Emergency department workplace interruptions: are emergency physicians “interrupt-driven” and “multitasking”?. Acad Emerg Med.

[10] Chisholm CD, Weaver CS, Whenmouth L (2011). A task analysis of emergency physician activities in academic and community settings. Ann Emerg Med.

[11] Sherbino J, Van Melle E, Bandiera G (2014). Education scholarship in emergency medicine part 1: innovating and improving teaching and learning. CJEM.

[12] Boyer EL (1990). Scholarship reconsidered: priorities for the professoriate.

[13] Steinert Y, Snell L, Sherbino J, Frank JR (2011). Educational innovation and scholarship: from curriculum design to implementation. Educational design: a CanMEDS guide for the health professions.

[14] Shulman L (1999). The scholarship of teaching. Change.

[15] Glassick CE (1997). Scholarship assessed: evaluation of the professoriate.

[16] Van Melle E, Lockyer J, Curran V (2014). Toward a common understanding: supporting and promoting education scholarship for medical school faculty. Med Educ.

[17] Glassick CE (2000). Boyer’s expanded definitions of scholarship, the standards for assessing scholarship, and the elusiveness of the scholarship of teaching. Acad Med.

[18] Medical Milestones The British Journal of Medicine Website.

[19] Lang ES, Wyer P, Tabas JA (2010). Educational and research advances stemming from the Academic Emergency Medicine consensus conference in knowledge translation. Acad Emerg Med.

[20] GL’99 Conference Program (1999). Fourth International Conference on Grey Literature: New Frontiers in Grey Literature.

[21] Sherbino J, Arora VM, van Melle E A Definition and Criteria for Social Media-based Scholarship in Health Professions Education: Results from the International Conference on Residency Education. Postgrad Med J.

[22] Nickson CP, Cadogan MD (2014). Free Open Access Medical education (FOAM) for the emergency physician. Emerg Med Australas.

[23] Biros MH, Barsan WG, Lewis RJ (1998). Supporting emergency medicine research: developing the infrastructure. Acad Emerg Med.

[24] Bhanji F, Cheng A, Frank JR (2014). Education scholarship in emergency medicine part 3: a “how-to” guide. CJEM.

[25] Sherbino J, Frank JR, Snell L (2014). Defining the key roles and competencies of the clinician-educator of the 21st century: a national mixed-methods study. Acad Med.

[26] Coates WC, Lin M, Clarke S (2012). Defining a core curriculum for education scholarship fellowships in emergency medicine. Acad Emerg Med.

[27] Yarris LM, Coates WC, Lin M (2012). A suggested core content for education scholarship fellowships in emergency medicine. Acad Emerg Med.

[28] Love JN, Coates WC, Santen SA (2009). The MERC at CORD Scholars Program in medical education research: a novel faculty development opportunity for emergency physicians. Acad Emerg Med.

[29] Confessore SJ (1997). Building a Learning Organization: Communities of Practice, Self-Directed Learning, and Continuing Medical Education. J Contin Educ Health Prof.

[30] Sherbino J, Snell L, Dath D (2010). A national clinician-educator program: a model of an effective community of practice. Med Educ Online.

[31] Farrell SE, Digioia NM, Broderick KB (2004). Mentoring for clinician-educators. Acad Emerg Med.

[32] Atasoylu AA, Wright SM, Beasley BW (2003). Promotion criteria for clinician-educators. J Gen Intern Med.

[33] Reed DA, Cook DA, Beckman TJ (2007). Association between funding and quality of published medical education research. JAMA.

[34] Bandiera G, Leblanc C, Regehr G (2014). Education scholarship in emergency medicine part 2: supporting and developing scholars. CJEM.

[35] Frank JR, Snell LS, Cate OT (2010). Competency-based medical education: theory to practice. Med Teach.

[36] Core Entrustable Professional Activities for Entering Residency (2014). Faculty and Learners’ Guide.

[37] Chan T, Sherbino J, McMAP Collaborators (2015). The McMaster Modular Assessment Program (McMAP): A Theoretically Grounded Work-Based Assessment System for an Emergency Medicine Residency Program. Acad Med.

[38] Asch DA, Epstein A, Nicholson S (2007). Evaluating medical training programs by the quality of care delivered by their alumni. JAMA.

[39] Cheston CC, Flickinger TE, Chisolm MS (2013). Social media use in medical education: a systematic review. Acad Med.

[40] Thoma B, Chan TM, Paterson QS (2015). Emergency Medicine and Critical Care Blogs and Podcasts: Establishing an International Consensus on Quality. Ann Emerg Med.

[41] Pillow MT, Hopson L, Bond M (2014). Social media guidelines and best practices: recommendations from the council of residency directors social media task force. West J Emerg Med.

[42] Thoma B, Sanders JL, Lin M (2015). The social media index: measuring the impact of emergency medicine and critical care websites. West J Emerg Med.

[43] Purdy E, Thoma B, Bednarcyzk J (2015). The use of free online educational resources by Canadian emergency medicine residents and program directors. CJEM.

[44] Mallin M, Schlein S, Doctor S (2014). A survey of the current utilization of asynchronous education among emergency medicine residents in the United States. Acad Med.

[45] Pusic MV, Boutis K, Hatala R (2015). Learning Curves in Health Professions Education. Acad Med.

[46] Forsetlund L, Bjørndal A, Rashidian A (2009). Continuing education meetings and workshops: effects on professional practice and health care outcomes. Cochrane Database Syst Rev.

[47] Lingard L, Espin S, Evans C (2004). The rules of the game: interprofessional collaboration on the intensive care unit team. Crit Care.

